# Bioactive Coatings Formed on Titanium by Plasma Electrolytic Oxidation: Composition and Properties

**DOI:** 10.3390/ma13184121

**Published:** 2020-09-16

**Authors:** Dmitry V. Mashtalyar, Konstantine V. Nadaraia, Andrey S. Gnedenkov, Igor M. Imshinetskiy, Mariia A. Piatkova, Arina I. Pleshkova, Evgeny A. Belov, Valeriia S. Filonina, Sergey N. Suchkov, Sergey L. Sinebryukhov, Sergey V. Gnedenkov

**Affiliations:** 1 Institute of Chemistry, Far Eastern Branch of the Russian Academy of Sciences, 690022 Vladivostok, Russia; madiva@inbox.ru (D.V.M.); asg17@mail.com (A.S.G.); igorimshin@gmail.com (I.M.I.); belov_eal@mail.ru (E.A.B.); filonina.vs@gmail.com (V.S.F.); suchkov.sn@students.dvfu.ru (S.N.S.); sls@ich.dvo.ru (S.L.S.); svg21@hotmail.com (S.V.G.); 2 School of Biomedicine, Far Eastern Federal University, 690091 Vladivostok, Russia; piatkova.mariia.al@gmail.com (M.A.P.); pleshkova.ai@students.dvfu.ru (A.I.P.)

**Keywords:** titanium, plasma electrolytic oxidation, biocompatible coatings, protective coatings, calcium phosphate, simulated body fluid, minimum essential medium, corrosion, wear, hydrophilicity

## Abstract

Bioactive coatings on VT1-0 commercially pure titanium were formed by the plasma electrolytic oxidation (PEO). A study of the morphological features of coatings was carried out using scanning electron microscopy. A composition of formed coatings was investigated using energy-dispersive spectroscopy and X-ray diffractometry analysis. It was shown that PEO-coatings have calcium phosphate in their composition, which increases the bioactivity of the surface layer. Electrochemical properties of the samples were studied by potentiondynamic polarization and electrochemical impedance spectroscopy in different physiological media: simulated body fluid and minimum essential medium. The data of electrochemical studies indicate more than 15 times decrease in the corrosion current density for the sample with coating (5.0 × 10^−9^ A/cm^2^) as compared to the bare titanium (7.7 × 10^−8^ A/cm^2^). The formed PEO-layers have elastoplastic properties close to human bone (12–30 GPa) and a lower friction coefficient in comparison with bare metal. The wettability of PEO-layers increased. The contact angle for formed coatings reduced by more than 60° in comparison with bare metal (from 73° for titanium to 8° for PEO-coating). Such an increase in surface hydrophilicity contributes to the greater biocompatibility of the formed coating in comparison with commercially pure titanium. PEO can be prospective as a method for improving titanium surface bioactivity.

## 1. Introduction

Currently, titanium and titanium alloys are widely used in implant surgery for orthopedic prostheses production [1,2,3]. The use of titanium is due to a number of its unique properties, such as high strength and corrosion resistance, leading to significant bioinertness of this material [4,5,6]. However, titanium alloys have several significant drawbacks. Thus, the implant surface requires additional, sometimes extremely complex processing to enhance osseointegration and increase bone adhesion. Moreover, a poor tribological performance of titanium alloys can lead to damage to adjacent tissues under heavy loads [1,4,7,8,9,10]. In connection with the above disadvantages of titanium alloys and the increasing necessity for the quality of implant materials, there is a need for improving the tribological characteristics, as well as the degree of osseointegration of orthopedic products made from this material. One of the possible ways in which to solve the problems mentioned above is the formation on the surface of a titanium implant of a functional coating possessing a required set of characteristics [11,12,13,14,15,16]. Today, there are several ways to form biocompatible and/or bioactive coatings on implants by deposition of the hydroxyapatite or other calcium phosphates on their surface. These methods include techniques such as ion beam-assisted deposition, plasma spray deposition, electrophoretic deposition, pulsed laser physical vapor deposition, plasma electrolytic oxidation, and magnetron sputtering deposition, etc. Each of these methods has its own advantages and disadvantages. The presented methods were described in more detail in the work of Paital and Dahotre [17]. Thus, one of the methods of coatings application used to increase wear resistance is thermal oxidation, which allows forming a durable rutile layer and an oxygen-saturated titanium sublayer on the interface coating/substrate that significantly improves tribological properties details as a whole [18,19,20,21,22]. However, taking into account the composition of the thermal oxide layer, the biocompatibility of this coating remains in question. In this regard, considerable attention is currently being paid to methods for forming coatings containing calcium phosphate compounds [12,23,24,25]. Among these methods, plasma electrolytic oxidation (PEO) can be distinguished, which allows one, due to varying the electrolyte composition and formation modes, to significantly change the composition and structure of the synthesized surface layer [26,27,28,29,30,31,32,33,34,35,36,37,38,39]. Thus, Ahounbar et al. formed biocompatible coatings on titanium scaffolds using calcium acetate and trisodium phosphate electrolytes [37]. In another work, Huang et al. formed surface layers with biocompatibility and antibacterial ability using the PEO technique [31]. Previously, PEO-coatings with high corrosion and wear resistance were successfully obtained [40,41,42,43]. The PEO process operates at an essentially higher electric field strength in comparison with traditional anodizing [44]. As a result of this, plasma discharges take place on the surface of the treated sample, in the channel of these discharges, along with the oxidation process, and substances from the electrolyte are transported and interact with the substrate material. It should also be noted that coatings formed by the PEO method have a developed (convoluted) surface morphological structure; this can significantly increase the possibility of bonding of the body tissue and the implant [45,46]. One of the important advantages of the PEO technique is the possibility of changing the implant surface with the aim of forming coatings with elastoplastic properties (microhardness, Young’s modulus) close to human bone tissue; it can also reduce the chance of implant failure [47]. Note that today, other stringent requirements are also imposed on the surface of implants. One of these requirements is high corrosion resistance [3], which is associated, first of all, with the need to prevent the ingress of corrosion products into the human organism. In addition, an important parameter is the wettability of the implant surface. It is known that a high wettability provides the increase in the implant osseointegration [48].

The aim of our study was to evaluate the prospects of using coatings formed by the PEO method in biomedicine. This paper presents a method for the formation of calcium phosphate coatings on commercially pure titanium, and a study of the structural features and composition of the obtained surface layers, their wettability, mechanical and corrosion properties.

## 2. Materials and Methods

### 2.1. Samples

As materials for the studied samples, VT1-0 commercially pure titanium was used (Table 1).

To estimate the electrochemical and mechanical characteristics, as well as wettability, 30 × 15 × 1 mm^3^ samples were used.

Before coating formation, in order to make the surface even, all samples were processed with sanding paper sequentially with a decrease in grain size down to 3 μm (the average roughness *R*_a_ determined using a Surtronic 25 profilometer (Taylor Hobson Ltd., Leicester, UK) did not exceed 1.2 μm). Then, all samples were washed with distilled water and degreased with alcohol.

### 2.2. Formation of Coatings

The control of plasma electrolytic oxidation process, as well as the measurement of electrical parameters, were carried out using an automatic system coupled with a computer with the appropriate software. Plasma electrolytic oxidation (PEO) of the samples was carried out in an electrolyte containing 25 g/L calcium glycerophosphate (C_3_H_7_CaO_6_P), 5 g/L sodium fluoride (NaF), and 7 g/L sodium metasilicate (Na_2_SiO_3_) in a combined monopolar mode.

During the PEO, the voltage increased to 300 V at a sweep rate of 0.5 V/s in order to realize plasma microdischarges at the electrolyte/sample interface, which are necessary for the formation of the PEO-layer. The duration of the PEO process was 300 s. The duty cycle was 50%.

### 2.3. Morphology and Composition of Coatings

An EVO 40 scanning electron microscope (Carl Zeiss, Oberkochen, Germany) was used to study surface morphology and analyze cross-sections of the coatings. In addition, this microscope was equipped with an INCA X-act instrument (Oxford Instruments, Abingdon-on-Thames, UK) for energy dispersive spectroscopy (EDS). The content of elements in the coating was investigated by comparing and evaluating line intensities. Before analysis, a thin layer (100 nm) of Cr was sprayed onto the samples with the 5 × 5 × 1 mm^3^ size (the use of a small sample is due to the dimensions of the scanning electron microscope chamber). The sprayed film provided sufficient electrical conductivity of the samples surface layer, which is necessary to prevent the formation and accumulation of electric charge on its surface. For the purpose of a more detailed study of the composition and structure of the coatings, we also evaluated the cross-sections of the coating.

The phase composition of the surface layers was studied in the “Far East Center for Structural Research” on a Rigaku multipurpose X-ray diffractometer (XRD, SmartLab, Tokyo, Japan) (Cu-*K*_α_ radiation) in the 2θ angles range of from 10° to 90° with a step of 0.02° and an exposure time of 1 s at each point.

### 2.4. Electrochemical Properties of Coatings

The electrochemical parameters of coatings formed on VT1-0 commercially pure titanium were studied by potentiodynamic polarization and electrochemical impedance spectroscopy (EIS) using the VersaSTAT MC electrochemical system (VMC-4) (Princeton Applied Research, Oak Ridge, TN, USA). The measurements were carried out in a three-electrode cell in two solutions: simulated body fluid (SBF) and minimum essential medium (MEM, Gibco^®^, Thermo Fisher Scientific, Waltham, MA, USA), prepared according to the protocols presented in [49,50], respectively, in order to determine the electrochemical properties of samples in contact with body fluid. The use of SBF is due to the proximity of its ionic composition to the ionic composition of human blood plasma (Table 2) [51]. MEM also imitates the inorganic composition of human blood plasma as well as organic composition to mimic the protein of mammalian cells [52]. Tests in MEM were performed at room temperature. The temperature for electrochemical measurements in SBF (37.5 ± 0.5 °C) was stabilized using a BD 23 incubator (BINDER, Tuttlingen, Germany). As a counter electrode, a niobium mesh coated with platinum was used. The reference electrode was a saturated calomel electrode (SCE). To standardize the results of studies, the exposed surface area of the samples was equal to 1 cm^2^.

Before starting electrochemical measurements, the samples were kept in solution for 60 min to achieve a steady state condition. The last potential value was used at potentiostatic EIS measurements. The sinusoidal signal had an amplitude of 10 mV (rms) during impedance measurements. Tests were carried out in the frequency range from 10^−2^ to 10^6^ Hz with a logarithmic sweep of 10 points per decade.

Potentiodynamic polarization test of samples with and without coatings were carried out with a potential sweep rate of 1.0 mV/s. The potential scan was carried out in the range from *E*_C_ − 0.25 V to *E*_C_ + 3.00 V.

The Levenberg–Marquardt method was used for fitting the experimental dependence of current density *I* on potential *E* [43,53,54,55]:

(1) $$I=I_{C}\times \left(10^{\frac{E-E_{C}}{\beta_{a}}}+10^{-\frac{\left(E-E_{C}\right)}{\beta_{c}}}\right).$$

This method makes it possible to obtain the best-fit values of corrosion potential *E*_C_, corrosion current density *I*_C_, the slopes of the cathodic *β*_c_ and anodic *β*_a_ polarization curves. The application of the Levenberg–Marquardt method ensured high accuracy of the calculations of corrosion parameters.

The polarization resistance *R*_P_ was determined in a separate experiment with potentiodynamic polarization of the sample in the potential region Δ*E* = *E*_C_ ± 20 mV with a scan rate of 0.167 mV/s, in which the linear dependence *I* = *f* (*E*) is observed.

Calculation of *R*_P_ values is carried out according to Equation (2):

(2) $$R_{P}=\frac{\Delta E}{\Delta I}$$

The impedance spectra presented in this work contain experimental data (marked with symbols) and theoretical curves obtained on the basis of the calculated equivalent electrical circuits (EEC) parameters and describing the experimental results with a high accuracy (the χ^2^ values are in the range (1.0 − 1.5) × 10^−4^).

In EEC, we used a constant phase element (CPE) instead of an ideal capacitance. The use of CPE is due to the heterogeneity of the systems under the study. CPE impedance was calculated using Equation (3):

(3) $$Z_{\mathrm{CPE}}=\frac{1}{Q\times {\left(j\times \omega \right)}^{n}}$$

where *ω* is the angular frequency (*ω =* 2*πf*), *j* is an imaginary unit, n is the exponential coefficient, and Q is the frequency independent constant.

### 2.5. Mechanical Properties of Coatings

The values of microhardness and elastoplastic properties of the studied coatings were obtained using a dynamic ultramicrohardnessmeter DUH–W201 (Shimadzu, Kyoto, Japan). Microhardness (*H*_μ_) was measured using the Berkovich indenter (Shimadzu, Kyoto, Japan). Evaluation and comparative analyses of the elastoplastic properties of coatings were carried out using the software “Shimadzu DUH Analysis Application v. 2.10” (Shimadzu, Kyoto, Japan).

Microhardness was determined as the ratio of the applied load to the contact area at this load. For the Berkovich tip, the calculation was carried out according to Equation (4):

(4) $$HU=\frac{F}{A_{s}\left(h\right)}\approx \frac{F}{26.43\times h_{1}^{2}}$$

(5) $$\mathrm{where}\;A_{s}\left(h\right)=\frac{3\times \sqrt{3}\times \tan\left(a\right)}{\cos\left(a\right)}\times h_{1}^{2}$$

where *F* is an applied load (mN), *h*_1_ is a penetration depth maximum of the indenter into the sample (μm), and α is a constant depending on the shape of the indenter.

The applied load in all measurements was 100 mN, the loading rate was 13.23 mN/s, and the retention time of the maximum load was 5 s. The tests were carried out at a temperature of 22–24 °C.

The tribological tests of the samples were carried out using a Tribometer TRB-S-DE device (CSM Instruments, Switzerland). The tests were carried out at 22–24 °C with a sliding speed of 25 mm/s. The tribological behavior of the coatings was studied under dry conditions and a load of 10 N. A corundum ball (α-Al_2_O_3_) was used as a counterbody. The number of wear cycles was 400.

### 2.6. Wettability of Coatings

The wettability of the obtained coatings was studied by the method of sessile drop using a DSA100 device (Krüss, Hamburg, Germany). The sessile drop method was used to measure the optical contact angle (CA) and evaluate the wetting properties of a localized solid surface area. In this method, the angle between the baseline of the drop and the tangent to the boundary of the drop was measured [56].

SBF and MEM were used as test liquids. The drop volume was equal to 2 μL. In order to obtain reliable data, 5 drops were applied to the surface of the studied samples. Additionally, in each experiment, five samples with different types of surface treatment were used.

## 3. Results and Discussion

### 3.1. Morphology and Composition of Coatings

**Figure 1:**
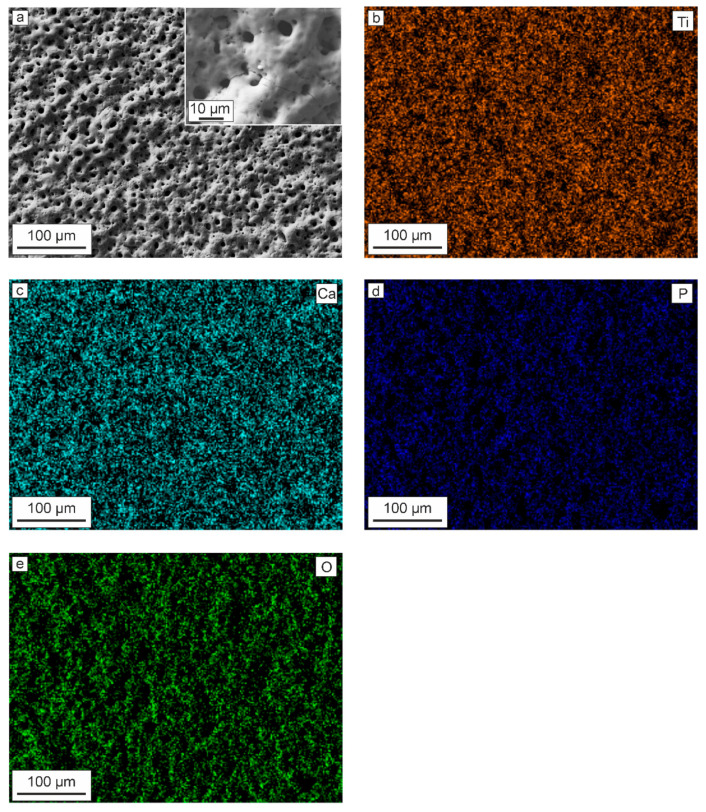
SEM-image of the (**a**) PEO-coating and a map of the elements distribution: (**b**) titanium, (**c**) calcium, (**d**) phosphorus, and (**e**) oxygen.

Based on the analysis of scanning electron microscopy data, it can be concluded that the formed layers have a surface morphology that is typical for PEO-coatings (Figure 1a).

There are a large number of pores and microdefects on the surface, which are the result of plasma discharges and gas evaporation during the PEO process, as well as a sharp cooling of the breakdown zone to the electrolyte temperature after attenuation of the plasma discharge [57]. It is well known that the developed (rough and uneven) surface has a positive effect on cell proliferation [48,58], including due to increasing the real contact area between the implant and body tissue. Analysis of the EDS data indicates the presence of such elements as Ti, Ca, P, and O in the coating composition (Figure 1b–d, Table 3). The presence of these elements, with the exception of titanium, which is part of the substrate, is a consequence of the transport of substances in the channel of plasma microdischarges from the electrolyte to the processed sample and further interaction between them.

**Figure 2:**
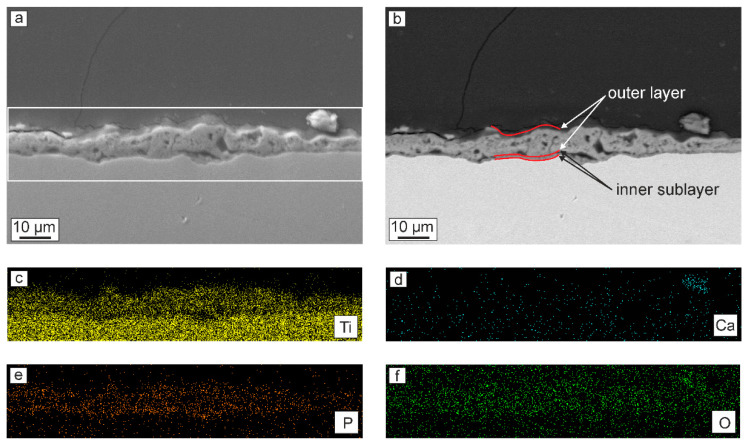
Cross-sectional SEM-images of the PEO-coating in (**a**) secondary and (**b**) backscattered electrons and a map of the elements distribution: (**c**) titanium, (**d**) calcium, (**e**) phosphorus, and (**f**) oxygen.

Analysis of the cross-section of the PEO-coating indicates its double-layer structure (Figure 2). The coating contains an inner thin non-porous sublayer with a thickness of about 1 μm and an outer porous layer with a thickness of about 8–10 μm (Figure 2b). The whole thickness of the coating does not exceed 12 μm (Figure 2a,b). The obtained results are in good agreement with the literature data [6,32,59]. The uneven distribution of Ti on the EDS map is a consequence of the capture of a part of the titanium substrate during the analysis (Figure 2a,c), as well as the presence of pores in the outer part of the coating (Figure 2b). Based on the EDS analysis of the cross-section (Figure 2), it can be concluded that the distribution of such elements as Ca, P, and O is uniform over the thickness of the coating (Figure 2c–e). Such a uniform distribution of elements is a consequence of the even distribution of plasma microdischarges on the surface of the sample during PEO. The obtained results are in good agreement with the results obtained earlier in [1,47].

Note that the presence in the coating composition in large quantities of such elements as Ca and P (Figure 1 and Figure 2, Table 3) will undoubtedly increase the possibility of osseointegration of the material surface. Moreover, the Ca/P ratio obtained in the PEO-layer is 1.17, which is close to the ratio of these elements in bone tissue: 1.67 [60,61].

**Figure 3:**
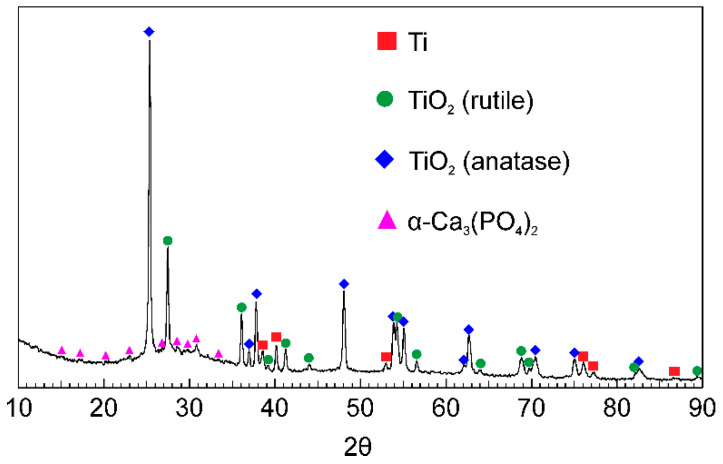
XRD patterns PEO-coating.

Analysis of the diffraction patterns of samples with PEO-coatings indicated the presence of TiO_2_ in the rutile and anatase forms as well as α-Ca_3_(PO_4_)_2_ (Figure 3). All these compounds were formed during the plasma-chemical synthesis of the surface layer substances. The formation of TiO_2_ in two forms is due to the mechanism of the PEO process. The temperature of plasma microdischarges, sometimes reaching 10,000 K, significantly exceeds the temperature values necessary for the formation of the rutile phase. However, the short lifetime of microdischarges (about 100 μs) leads to a sharp cooling of the breakdown zone to the electrolyte temperature, thereby reducing the probability of rutile formation [62,63]. Thus, both modifications of TiO_2_ are simultaneously present in the composition of PEO-coatings (Figure 3). The presence of a halo at small angles (Figure 3) may be due to the presence of amorphous oxides of variable composition in the coating.

The presence of α-Ca_3_(PO_4_)_2_ in the surface layer of the samples (Figure 3) allows us to conclude about the increased osseointegration of PEO-coatings due to the similarity of their composition to the mineral composition of human bone tissue, in which α-Ca_3_(PO_4_)_2_ is also presented [17]. Additionally, it is well known that α-Ca_3_(PO_4_)_2_ promotes the formation of hydroxyapatite Ca_10_(PO_4_)_6_(OH)_2_ in human biological fluids [17]. Thus, a calcium phosphate PEO-coating applied to a titanium implant can form bone growth centers, significantly accelerating the healing time of an injury.

### 3.2. Electrochemical Properties of Coatings

#### 3.2.1. Tests in SBF

**Figure 4:**
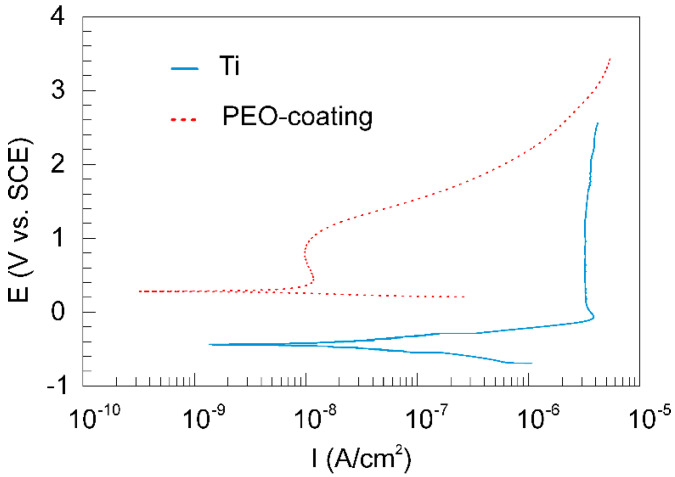
Polarization curves for uncoated sample and sample with a PEO-coating in SBF.

Comparative analysis of the corrosion behavior of the samples with and without protective coating in SBF was performed in this work. Potentiodynamic polarization data indicate an improvement in the corrosion properties of the samples after applying a PEO-layer to their surface. Thus, there is a shift of the corrosion potential E_C_ to the positive region for PEO-coating in comparison with the uncoated material (Figure 4, Table 4), which is a consequence of the formation of a protective layer on the surface. Additionally, for PEO-coatings, a decrease in the corrosion current density I_C_ and an increase in the polarization resistance R_P_ by more than twofold are observed in comparison with bare titanium (Figure 4, Table 4).

Generally, the presented data (Figure 4, Table 4) allow concluding that the protective properties of PEO-coatings are greater in comparison with untreated VT1-0 titanium in SBF. Evaluation of the polarization curves presented in Figure 4 indicates certain differences in the corrosion process for samples with and without a PEO-coating. For bare VT1-0 titanium, the fast increase in the current density at −0.3 V is the result of the pitting formation and partial destruction of the natural oxide film (Figure 4). Then, with an increase in the potential of more than −0.1 V, titanium is passivated (Figure 4). For the PEO-coating, the beginning of the passivation process in the anode region is observed at potential values of about 0.4 V, and there is no sharp increase in I values (Figure 4). This is due to the presence of a compact, almost non-porous sublayer in the structure of the PEO-coating (Figure 2), which increases the corrosion resistance of the sample.

**Figure 5:**
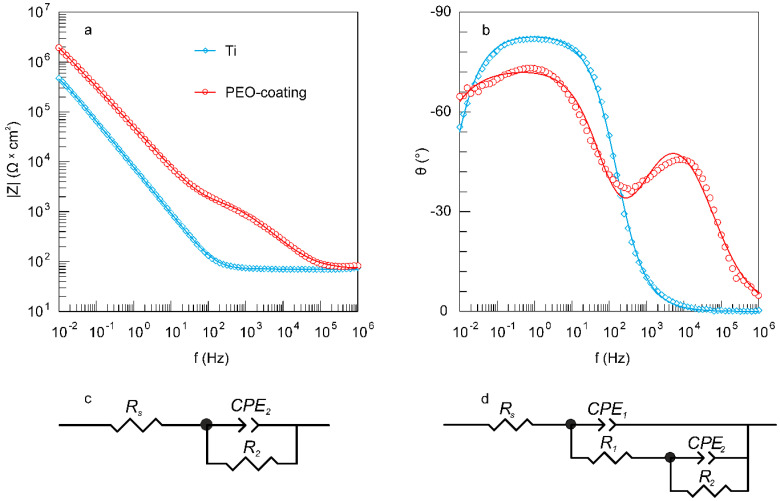
Bode plots (dependence of (**a**) impedance modulus |Z| and (**b**) phase angle θ on frequency f) for uncoated sample and sample with a PEO-coating and equivalent electrical circuits used to fit the experimental impedance spectra: (**c**) one–R–CPE–circuit, (**d**) two–R–CPE–circuit. Impedance spectra contain experimental data (scatter plot marked by symbols) and theoretical fitting curves (lines), which simulate the experimental results by means equivalent electrical circuits. Test liquid is SBF.

The data of electrochemical impedance spectroscopy presented in Figure 5a,b in the form of Bode plots, indicate a positive effect of the formed PEO-layer on the protective properties of the sample. The values of the impedance modulus measured at low frequencies |Z|_f = 0.01 Hz_ for the PEO-coating are more than four times higher than those for the bare VT1-0 titanium (Figure 5a, Table 5). Thus, coating formation significantly reduces the possibility of charge transfer at the electrolyte/sample interface, which, in turn, reduces the possibility of corrosion processes. Note that the EIS data (Figure 5a, Table 5) are in good agreement with the data obtained by the potentiodynamic polarization (Figure 4, Table 4).

On the graph of the phase angle θ dependence on the frequency f (Figure 5b) for titanium untreated by the PEO, there is one time constant in the medium and low frequencies (approximately, from 10^−2^ to 10^2^ Hz) (Figure 5b). The appearance of this constant is due to the presence of the natural oxide film (mainly TiO_2_) on the titanium surface. This spectrum can be fitted using an EEC with one R_2_–CPE_2_-circuit, where R_2_ is the charge transfer resistance and CPE_2_ is the capacity of the natural oxide film (Figure 5c).

Two time constants are observed in the spectrum of the sample with PEO-layer. First of which is located in the high-frequency range with a minimum of about 8 × 10^3^ Hz, and the second one is in the mid- and low-frequency range with a minimum of 0.8 Hz (Figure 5b). The presence of these time constants is due to the structure of the PEO-layer, namely, the presence in the coating morphology structure of the outer porous layer (bend in the high frequency region) and the inner non-porous sublayer (bend in the medium and low frequencies) (Figure 2). This, in turn, is in good agreement with both our previous studies [43] and literature data [32]. The presented spectrum is described using a two-R–CPE-circuit EEC (Figure 5d). In this EEC, the R_1_–CPE_1_ element takes into account the porous, and the R_2_–CPE_2_ element describes the non-porous parts of the PEO-coating.

Table 5 shows the calculated parameters of the circuit elements for the corresponding EEC (Figure 5). Based on the analysis of the presented data (Table 5), it can be concluded that the parameter R_2_ increases and the Q_2_ values decrease by one order of magnitude for samples with a PEO-layer in comparison with bare material. This is a consequence of the significantly greater thickness of the inner sublayer of the PEO-coating in comparison with a thin film of natural oxide on untreated titanium samples. We also note that the values of n_2_ decrease for samples after processing them with the PEO-method (Table 5), which is due to a decrease in the homogeneity of the inner sublayer of the PEO-coating in comparison with the oxide film on the uncoated titanium surface.

Analysis of the results of comparing the parameters of the R_1_–CPE_1_- and R_2_–CPE_2_-circuit for the PEO-coating indicates the different contributions of the porous and non-porous layers to the charge transfer process at the electrolyte/PEO-coating interface. Thus, the resistance of the inner sublayer R_2_ is almost four orders of magnitude higher than the value of R_1_ (resistance of the outer porous layer), which leads to a more significant contribution of the first one to the corrosion properties of the coating (Table 5). Moreover, a higher heterogeneity of the outer porous layer in comparison with a non-porous one is also indicated by values of n_1_, which are smaller than those for n_2_ (Table 5). Thus, the data of electrochemical impedance spectroscopy confirm the conclusions made earlier during the evaluation of the results of scanning electron microscopy: the presence of a double-layer structure in the PEO-coating (Figure 2 and Figure 5b, Table 5).

#### 3.2.2. Tests in MEM

**Figure 6:**
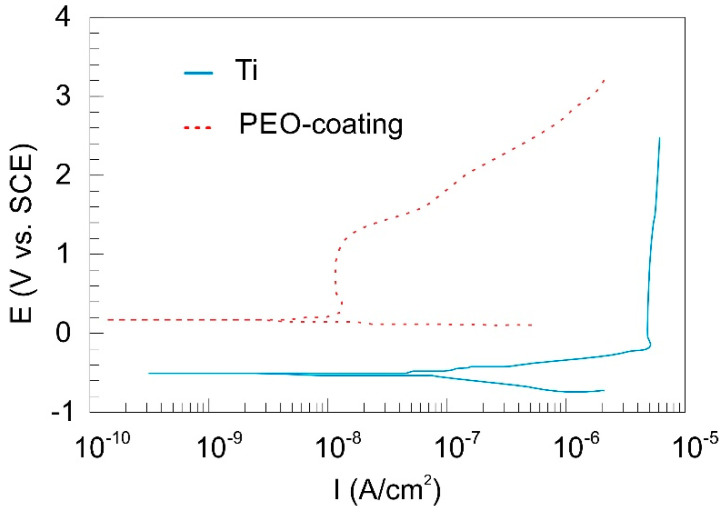
Polarization curves for uncoated sample and sample with a PEO-coating in MEM.

Dissimilarity of the studied samples’ corrosion behavior is also observed during the research in minimum essential medium. Based on the analysis of obtained polarization curves (Figure 6, Table 4), it is revealed that, in comparison with an uncoated titanium, the corrosion potential E_C_ of PEO-treated sample takes on positive values. Moreover, formation of the PEO-layer contributes to a decrease in corrosion current density I_C_ of more than a one order of magnitude (Figure 6, Table 4). This set of results obviously indicates that the formation of the PEO-coating contributes to the improvement of surface protective properties. During the comparative evaluation of obtained polarization curves (Figure 6), the difference in electrochemical behavior of studied surface layers is observed. The behavior of the uncoated sample at anodic polarization is characterized by a sharp increase in the current density from E_C_ to approximately −0.2 V, which is correlated with a pitting formation process at potential near E_C_. A rapid growth in potential from −0.1 V, followed by non-changing current values signify the passivation of titanium surface. For the PEO-treated sample, the passivation starting point is shifted to a positive area (nearly 0.4 V) and no rapid decline in corrosion current density is observed.

**Figure 7:**
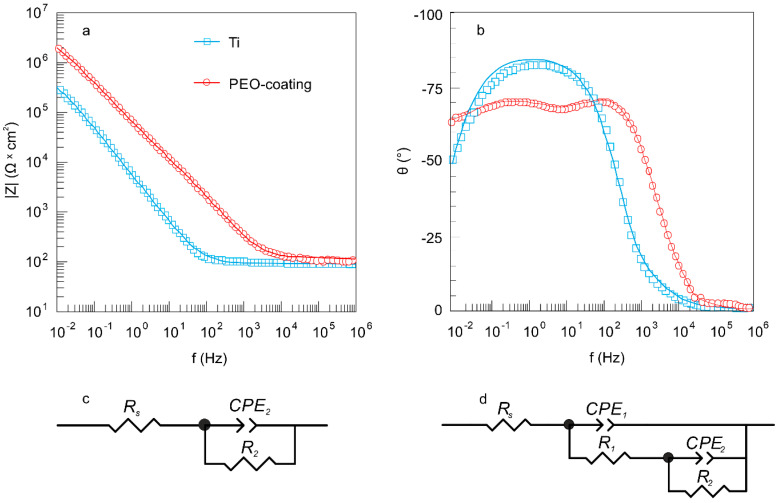
Bode plots (dependence of (**a**) impedance modulus |Z| and (**b**) phase angle θ on frequency f) for uncoated sample and sample with a PEO-coating and equivalent electrical circuits used to fit the experimental impedance spectra: (**c**) one–R–CPE–circuit, (**d**) two–R–CPE–circuit. Impedance spectra contain experimental data (scatter plot marked by symbols) and theoretical fitting curves (lines), which simulate the experimental results by means of equivalent electrical circuits. Test liquid is MEM.

The results of electrochemical impedance spectroscopy study in MEM are presented in Figure 7 and Table 5. They are in coherence with the ones obtained by potentiodynamic polarization and previous studies in SBF, presented in paper.

According to the obtained data, values of impedance modulus, measured at lowest frequency |Z|_f = 0.01 Hz_ for the PEO-treated sample are almost eight times higher than those for the uncoated sample (Figure 7, Table 5). Based on the results, it can be concluded that plasma electrolytic surface treatment is followed by the reduction in corrosion processes’ possibility due to the difficulty of discharge transfer on a solid (sample) and liquid (electrolyte) interface. It should be noted that similar corrosion behavior was found for the sample studied in SBF. On the phase angle θ–frequency dependence Bode plot (Figure 7b), bare VT1-0 titanium is characterized by a single time constant at medium and low frequencies. This spectrum corresponds to a formed passive oxide film, consisting mainly of titanium dioxide and can be fitted with EEC with one R–CPE-circuit (Figure 7c). In such a case, the R_2_ component defines the charge transfer resistance and the CPE_2_ component characterizes the capacity of an oxide film (Table 5). The impedance spectrum of the sample with PEO-layer is characterized by the presence of two time constants and delineates by EEC with two R–CPE-circuit with a series-parallel connection (Figure 7d), which was previously described in Section 3.2.1. An observed configuration occurs due to the structure of a surface layer, formed as a result of PEO. Thus, the R_1_–CPE_1_-circuit with the minimum of 10^2^ Hz (high-frequency range) characterizes the porous layer of the PEO-coating, and a non-porous sublayer is described with the R_2_–CPE_2_-circuit with the minimum of 0.5 Hz (low-frequency range) (Table 5).

All the calculated EES parameters presented in Table 5 for the samples in MEM are in good agreement with those for the specimens in SBF. Higher values of R_1_ for the sample in MEM is a result of MEM products’ accumulation in coating pores. These products block the pathway for the corrosive medium to the non-porous part of the protective layer. Therefore, the resistance of the porous layer increases as compared to one for the sample in SBF. This result is also confirmed by the higher value of n_1_ (0.90) as compared to one calculated for the specimen in simulated body fluid (0.73). This indicates the increase in the PEO-layer homogeneity as a result of pores sealing.

### 3.3. Mechanical Properties of Coatings

**Figure 8:**
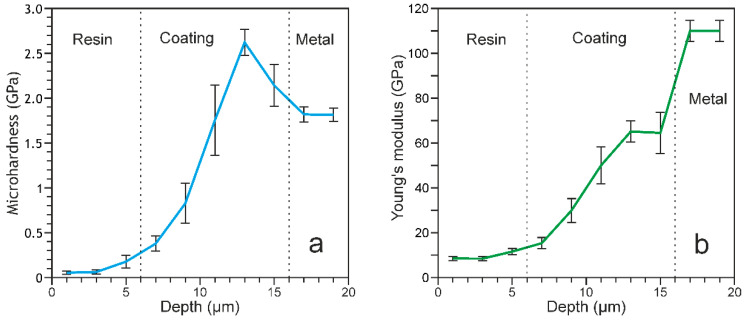
Distribution (**a**) microhardness and (**b**) Young’s modulus by thickness for PEO-coating.

To study the mechanical properties of the PEO-treated samples, the microhardness test was carried out on cross-sections within the coating thickness (Figure 8a). According to the EIS data and SEM-images of cross-sections (Figure 2, Figure 5 and Figure 7, Table 5), the outer layer of the coating has the highest porosity, which significantly affects its microhardness (0.2–0.3 GPa) (Figure 8a). When approaching a metal substrate, a decrease in the coating porosity, and as a result, an increase in the microhardness values were observed. The highest values were obtained for the non-porous sublayer and attain (2.6 ± 0.1) GPa (Figure 8a), which is actually 1.5 times higher than that of commercially pure VT1-0 titanium ((1.8 ± 0.1) GPa). The main reason for increasing the coating hardness is the presence of titanium dioxide in a rutile modification (Figure 3).

The change in the values of Young’s modulus over the thickness of the PEO-coating is the same as the change in microhardness. The elastic modulus values vary from 12 ± 2 to 65 ± 5 GPa, which is significantly lower than that for a titanium substrate—110 ± 5 GPa (Figure 8b). Note that the obtained elastic modulus values of the upper region of the coatings are 12–30 GPa (Figure 8b), which is close to the values of the elastic modulus of human bones (10–30 GPa) [64,65,66,67]. Thus, the trauma of the adjacent tissues to implant will reduce and the cell proliferation on the bioactive implant surface will increase. At the same time, a gradual increase in the microhardness and elastic modulus as it approaches the substrate material (Figure 8) allows the implant to maintain high strength properties, which will undoubtedly contribute to maintaining the patient’s activity.

**Figure 9:**
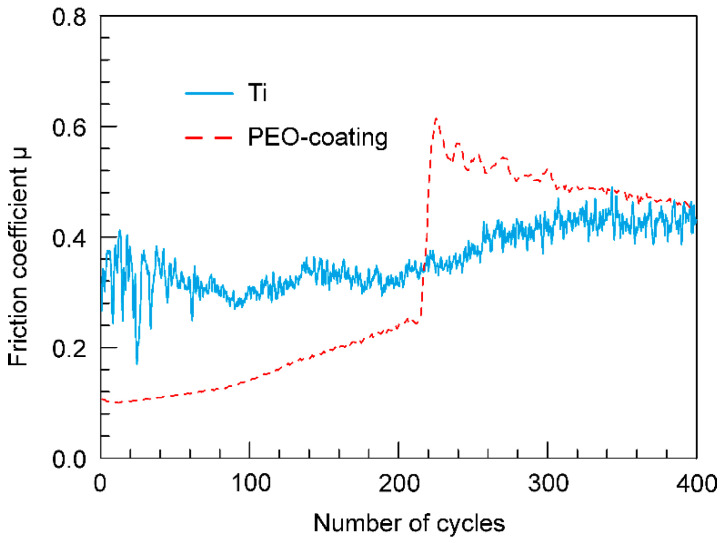
Dependence of the friction coefficient *μ* on the number of cycles for uncoated sample and sample with a PEO-coating.

Analysis of the data presented in Figure 9 indicates a significant effect of the modification of the surface layer on the tribological properties of the samples. Uncoated titanium is characterized by changes in the friction coefficient over a range from 0.3 to 0.4 (Figure 9). Apparently, this is due to the viscosity of the metal itself, and therefore, during the abrasion process, an uneven load distribution occurs, leading to changes in the friction coefficient (Figure 9). This is also due to an increase in the contact area as titanium abrades. In the case of a possible contact between the titanium implant and body tissues, such uneven loads can lead to the separation of the first one from the bone tissue.

In contrast to the uncoated sample, two abrasion stages can be indicated for the PEO-coating. At the first stage, the duration of which is about 220 cycles, the values of the friction coefficient do not exceed 0.25 (Figure 9). At this stage, the PEO-coating is gradually abraded, while the particles of the outer porous layer formed during its destruction work as an abrasive material under the permanent increase in the area of contact of corundum ball with coating (Figure 9). Furthermore, in the second stage, a sharp increase in the friction coefficient up to 0.64 is observed (Figure 9) due to the partial destruction of the PEO-coating until metal is exposed on some parts of the track. Then, the friction coefficient gradually decreases, approaching the values characteristic for a metal (Figure 9), which is a result of rubbing of the tribocouple and removing the particles of destroyed coating from the contact zone through friction. Thus, the PEO-coating can reduce the values of the friction coefficient in the first part of the wear track distance. This can help to stabilize the load in the contact zone between the body tissues and the implant, which, in turn, will increase the bone-to-implant contact.

### 3.4. Wetting of Coatings

**Figure 10:**
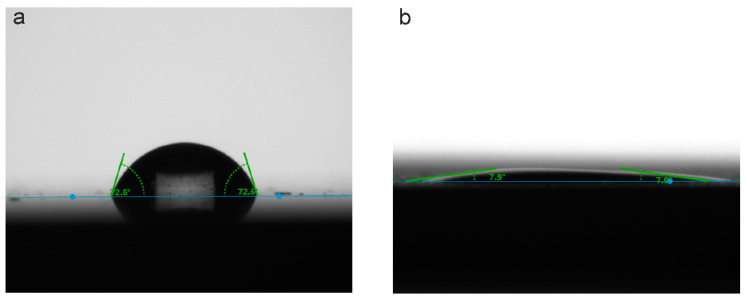
Drops form and contact angle values for uncoated sample (**a**) and sample with a PEO-coating (**b**) Test liquid is SBF.

Evaluation of the wettability of samples with different types of surface treatment using SBF as a test solution indicates a significant difference in their hydrophilic properties (Figure 10, Table 6).

Thus, the surface of the titanium alloy has hydrophilic properties (CA = 70 ± 5°) (Figure 10, Table 6). After applying the PEO-layer to the titanium surface, the wettability increases significantly, and the CA decreases to 8° ± 1°, which allows us to characterize this coating as near-superhydrophilic [48]. We also note that we were able to measure the CA values only in the first second after applying the drop to the surface, because, further, it completely spread, and the evaluation of the contact angle was not possible (Video S1, Supplementary Material).

**Figure 11:**
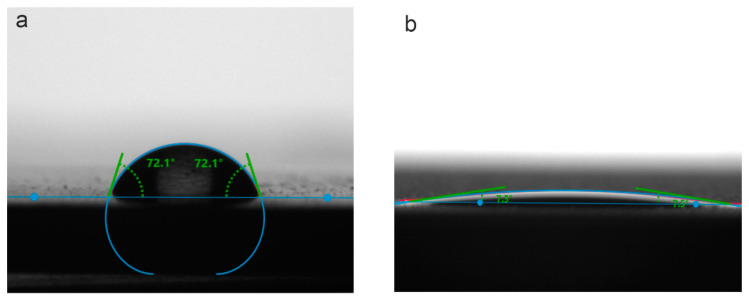
Drops form and contact angle values for uncoated sample (**a**) and sample with a PEO-coating (**b**) Test liquid is MEM.

Additionally, the wettability of the studied surface layers was evaluated using MEM as a test liquid. The obtained results are similar to those for the specimen studied using SBF. Analysis of the data presented in Figure 11 and Table 6 highlights the quite low contact angle values of the bare VT1-0 titanium surface (CA = 73 ± 2°). The following PEO treatment of the metal surface (just like for the specimen in SBF) contributes to a sharp decrease in contact angle values, respectively increasing surface wettability (CA = 8 ± 2°) (Figure 11, Table 6).

High wettability is an important property for the implant surface. Such a surface increases the bone-to-implant contact, which leads to rapid and long-term osseointegration [48]. Thus, in addition to the coating composition, as well as the elastoplastic characteristics close to cortical bone, the obtained surface layer due to their developed surface (Figure 1 and Figure 2) showed high wettability in contact to physiological solutions, which also increases the probability of implant osseointegration, as well as fibroblast spreading. It makes such a surface modification technique highly suitable for bone tissue engineering.

## 4. Conclusions

Calcium phosphate coatings were obtained on commercially pure titanium by plasma electrolytic oxidation. The formed surface layer possesses mechanical and physico-chemical performance, which are fundamentally important for biomedicine. The study established the relationship between the composition, structure, and properties of coatings. Using the EDS method, it was found that both calcium and phosphorus are present in large amounts in the coating, the ratio of which is close to the ratio of these elements in bone tissue. Analysis of the diffraction patterns of PEO-coatings revealed the presence of α-Ca_3_(PO_4_)_2_ in the composition of the surface layers, which is also one of the main mineral components of human bone tissue and contributes to the formation of hydroxyapatite. PEO-coatings possess the high corrosion resistance in comparison with uncoated titanium, reducing the corrosion current density by more than 15 times. The microhardness and Young’s modulus of the formed coating are close to the ones for the cortical bone. The obtained PEO-layers possess high hydrophilic properties, which, in turn, can contribute to an increase in the bone-to-implant contact. Summarizing all the above, this enables one to conclude that PEO can be a prospective technique in implantology as a method of the surface treatment of titanium endoprostheses.

## Figures and Tables

**Table 1:** Elemental composition of the VT1-0 commercially pure titanium.

Element	wt.%
Fe	0.25
Si	0.12
C	0.07
O	0.12
N	0.04
H	0.01
Ti	Balance

**Table 2:** Ion concentration (mM) in human blood plasma and simulated body fluid (SBF).

Ion	SBF	Human Blood Plasma
Na^+^	142.0	142.0
K^+^	5.0	5.0
Mg^2+^	1.5	1.5
Ca^2+^	2.5	2.5
Cl^−^	103.0	103.0
HCO_3_^−^	10.0	27.0
HPO_4_^2−^	1.0	1.0
SO_4_^2−^	0.5	0.5

**Table 3:** Elemental composition of plasma electrolytic oxidation (PEO)-coatings.

Element	at. %
Ti	15.6
Ca	7.5
P	6.4
O	70.5

**Table 4:** Corrosion properties of investigated samples in SBF and minimum essential medium (MEM).

Sample	E_C_ (V vs. SCE)	I_C_ (A/cm^2^)	β_a_ (mV/Decade)	β_c_ (mV/Decade)	R_P_ (Ω × cm^2^)
**SBF**					
Bare VT1-0 titanium	−0.44	1.5 × 10^−8^	130	134	1.9 × 10^6^
With PEO-coating	0.28	4.2 × 10^−9^	181	50	4.1 × 10^6^
**MEM**					
Bare VT1-0 titanium	−0.54	7.7 × 10^−8^	168	217	5.3 × 10^5^
With PEO-coating	0.09	5.0 × 10^−9^	178	47	3.4 × 10^6^

**Table 5:** Calculated parameters of equivalent electrical circuits for samples with different types of surface treatment.

Sample	|Z|_f__= 0.__01 Hz_ (Ω × cm^2^)	R_1_ (Ω × cm^2^)	CPE_1_		R_2_ (Ω × cm^2^)	CPE_2_	
			Q_1_ (Ω^−1^ × cm^−2^ × s^n^)	n_1_		Q_2_ (Ω^−1^ × cm^−2^ × s^n^)	n_2_
**SBF**							
Bare VT1-0 titanium	4.7 × 10^5^	–	–	–	9.2 × 10^5^	2.3 × 10^−5^	0.93
With PEO-coating	1.9 × 10^6^	1.9 × 10^3^	1.4 × 10^−6^	0.73	1.2 × 10^7^	3.1 × 10^−6^	0.84
**MEM**							
Bare VT1-0 titanium	2.7 × 10^5^	–	–	–	4.3 × 10^5^	3.6 × 10^−5^	0.92
With PEO-coating	2.0 × 10^6^	2.0 × 10^4^	2.0 × 10^−6^	0.90	1.5 × 10^7^	1.8 × 10^−6^	0.71

**Table 6:** Wettability of the samples with different types of surface treatment.

Sample	Contact Angle (°)
**SBF**	
Bare VT1-0 titanium	70 ± 5
With PEO-coating	8 ± 1
**MEM**	
Bare VT1-0 titanium	73 ± 2
With PEO-coating	8 ± 2

## Supplementary Materials

The following are available online at https://www.mdpi.com/1996-1944/13/18/4121/s1, Video S1: Wettability of PEO-coating.
